# Finger Bending Sensing Based on Series-Connected Fiber Bragg Gratings

**DOI:** 10.3390/ma15103472

**Published:** 2022-05-12

**Authors:** Qijing Lin, Kun Yao, Na Zhao, Yunjing Jiao, Zelin Wang, Bian Tian, Libo Zhao, Gangding Peng, Zhuangde Jiang

**Affiliations:** 1 Collaborative Innovation Center of High-End Manufacturing Equipment, Xi’an Jiaotong University, Xi’an 710049, China; qjlin2015@xjtu.edu.cn; 2 State Key Laboratory of Mechanical Manufacturing Systems Engineering, Xi’an Jiaotong University, Xi’an 710049, China; zn2020@xjtu.edu.cn (N.Z.); yunjingjiao@stu.xjtu.edu.cn (Y.J.); wzl15086927209@stu.xjtu.edu.cn (Z.W.); t.b12@mail.xjtu.edu.cn (B.T.); libozhao@xjtu.edu.cn (L.Z.); zdjiang@xjtu.edu.cn (Z.J.); 3 School of Mechanical and Manufacturing Engineering, Xiamen Institute of Technology, Xiamen 361021, China; 4 Chongqing Key Laboratory of Micro-Nano Systems and Intelligent Sensing, Chongqing Academician Workstation, Chongqing 2011 Collaborative Innovation Center of Micro/Nano Sensing and Intelligent Ecological Internet of Things, Chongqing Technology and Business University, Chongqing 400067, China; 5 School of Electrical Engineering and Telecommunications, The University of New South Wales, Sydney 2052, Australia; g.peng@unsw.edu.au

**Keywords:** FBG, bending gesture sensing, smart glove, cubic spline interpolation

## Abstract

Smart wearable devices are occupying an increasingly important position in scientific research and people’s life fields. As an indispensable component of smart wearable devices, sensors play a crucial role in their sensing and feedback capabilities. In this paper, we investigate the bending gesture sensing for the most dexterous part of human anatomy, the finger. Based on series-connected fiber Bragg gratings (FBGs), recognition of finger bending posture is achieved by MATLAB modeling and the cubic spline interpolation.

## 1. Introduction

With the increasing demand for operations in harsh environments, such as high temperature environments, corrosive gas environments, and outer space environments, dexterous robotic hands are expected to replace humans to perform these operations. Therefore, the posture of the human hand needs to be studied to guide the precise operation of the dexterous robotic hand. As an effective method for recognizing hand posture, smart gloves have been studied in recent years. For example, the glove developed by Ulf Müller et al. [1] was used to relieve stresses for workers. The actuator of these gloves was made of memory alloy wires. Peter Zientara et al. [2] used the smart glove and glasses concept to develop a system to assist the visually impaired with a focus on grocery shopping. The smart glove proposed by Minglu Zhu et al. [3] achieved the function of haptic feedback by electronic sensors. The glove could generate different triboelectric signals when the fingers bent at different degrees. Wentao Dong et al. [4] used a stretchable smart glove to achieve the human machine interface. The smart glove was designed based on piezoresistive sensors to detection signals and control the movement of the robot’s fingers. Wei-Chieh Chuang et al. [5] proposed a finger gesture recognition system to collect sets of training and test based on flexible sensors. The glove integrated wireless system to realize wireless transmission of test data. James Connolly et al. [6] proposed a smart glove to measure knuckle movement of fingers to provide clinicians for the treatment of patients with rheumatoid arthritis. Finger motion was accurately measured by using multiple IMU inertial measurement unit sensors and control algorithms. Debeshi Dutta et al. [7] proposed a smart glove to provide timely estimation of grasp quality for better treatment of stroke patients. Moe Amanzadeh et al. [8] have presented a comprehensive critical review of the evolution trend and sensing principles of technologies used in the development of fiber optic shape sensors. Chandan Kumar Jha et al. [9] designed an FBG sensor-based glove to simultaneously monitor the flexure of ten finger joints and have shown the feasibility in recovery progress of stroke patients during the virtual rehabilitation therapy. In medical applications, Daniela Lo Presti et al. [10] have reviewed the FBG-based measuring systems, their principle of work, and their applications. Strengths, weaknesses, open challenges, and future trends have been discussed to highlight how FBGs can meet the demands of next-generation medical devices and healthcare system. Xiaoxue Han et al. [11] knitted a smart glove using a flat knitting machine. Hand postures were tested statically and dynamically by the glove. The development of edge computing technology has enabled smart gloves to be used in the internet of things (IoT). Ali Passian et al. [12] made a comprehensive and systematic summary of edge computing and related technologies, and made forward-looking predictions for the next generation of computing systems, which is very instructive.

Compared with electronic sensors, fiber optic sensors are resistant to harsh environments, insensitive to electromagnetic interference, multi-parameter sensing, and have flexible distribution sensing ability [13,14,15,16,17,18]. João Paulo Carmo et al. [19] proposed a fiber Bragg Grating (FBG)-based System for wearable devices. While embedded on flexible materials, the strain sensitivity of FBGs was increased and suitable for wearable garments. To detect human activity, Jingjing Guo et al. [20] proposed a strain sensor based on FBGs which were stretchable. This strain sensor can be used for real-time detection of breathing, vocalization, knuckle movements, and expressions. Martina Zaltieri et al. [21] proposed a wearable device embedded with FBG sensors to detect the flexions and extensions of lumbar when seated. To measure the bending angle of knees, Shweta Pant et al. [22] proposed a wearable device based on FBG sensors. The device converts the angular motion between the tibia and thigh into strain changes of a cantilever with FBG sensors. However, smart gloves based on multiplexed FBGs have rarely been studied.

In this paper, a smart glove with series FBGs is proposed for measuring the bending posture of fingers. Each knuckle has an FBG corresponding to it. By measuring the angle of each knuckle of the finger, using the method of linear fitting and coefficient correction, the measurement of the hand’s bending is achieved.

## 2. Materials and Methods

### 2.1. Materials

The optical fibers used in this study are single-mode fibers with a mold field diameter of 10 μm at a wavelength of 1550 nm and a cladding diameter of 125 μm. The Young’s modulus and the Poisson’s ratio of the optical fibers are 72 GPa and 0.15, respectively. The sponge used in this research has a Young’s modulus of 32.05 kPa and a Poisson’s ratio of 0.32. The fiber fusion splicer used in this research is a FITEL S178A. The optical demodulator used in this research is an MOI si155, with a wavelength range of 80 nm and a wavelength accuracy of less than 2 pm. ANSYS Workbench R15 software in this research is used to analyze force and deformation of the fiber.

### 2.2. Design of Sensing

**Figure 1:**
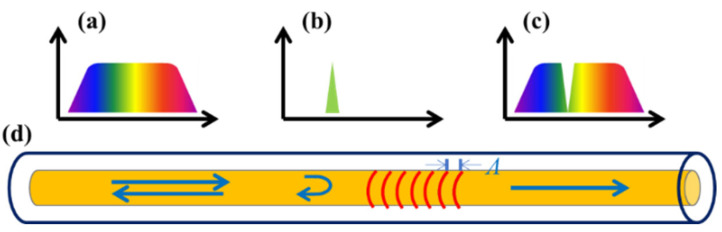
The schematic diagram of an FBG. (**a**) Incident spectrum. (**b**) Reflectance spectrum. (**c**) Transmission spectrum. (**d**) Optical fiber.

The FBG is obtained using laser or UV exposure to produce periodic refractive index changes in the direction along the axis of a quartz fiber. The schematic diagram of FBGs in series is shown in Figure 1. When broadband incident light (Figure 1a) passes through each FBG, light of a specific wavelength (Figure 1b–d) is reflected in its original optical path and light of other wavelengths (Figure 1c) passes through the FBGs. The reflected wavelength of each FBG is related to the interval of the grid and its effective refractive index, as described in Equation (1).

(1) $$\lambda =2n_{eff}\Lambda$$

where λ represents the wavelength of the reflected light; $n_{eff}$ represents the effective refractive index of the FBG; and Λ represents the interval of the FBG grid.

When the FBG is stretched in the axial direction, the wavelength of the reflected light changes due to the change of the grating length and the photoelastic effect. The wavelength change can be calculated by Equation (2):

(2) $$\Delta \lambda =\lambda \left\{1-\frac{n^{2}}{2}\left[(1-\upsilon )p_{12}-\upsilon p_{11}\right]\right\}=\lambda (1+\gamma )e_{z}$$

where λ represents the wavelength of the reflected light; n represents the refractive index of the fiber core; υ represents the Poisson’s ratio of the fiber; $p_{12}$ and $p_{11}$ represent the elasticity coefficients of the fiber; γ represents the effective elasticity factor; and $e_{z}$ represents the strain of the FBG in the axial direction.

**Figure 2:**
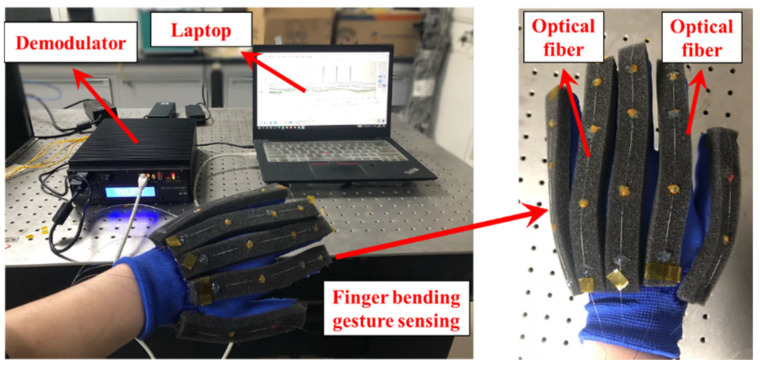
Fingers bending measured by FBGs.

Human fingers contain 2 to 3 knuckles. The bending of the finger contains the bending angle of each knuckle. In order to accurately measure the bending of the finger, it is necessary to measure the bending angle of every knuckle of the finger at the same time. FBGs connected in series are used to measure the bending of every knuckle. FBGs are pasted on a glove along the axis of every finger, as shown in Figure 2. Instead of pasted to the knuckles directly, the FBGs is pasted away from the knuckles to avoid damage to the FBGs by excessive bending of the fingers. To protect the optical fiber from being pulled off by the finger bending, a 1 cm thick sponge is pasted between the optical fiber and the glove for cushioning protection.

### 2.3. Analysis of Model

**Figure 3:**
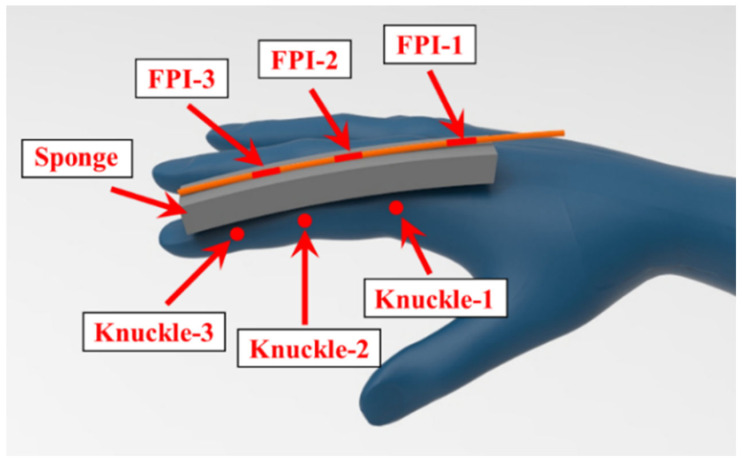
Diagram of pasted sponge and fiber.

**Figure 4:**
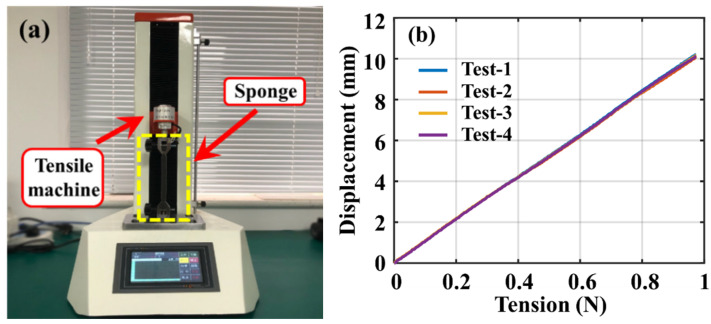
Measurement of the sponge parameters. (**a**) Tensile machine; (**b**) Mechanical properties of the sponge.

Take the index finger as an example, as shown in Figure 3. As the FBGs are connected in series in an optical fiber, the bending of any knuckle will have a pulling effect on all three FBGs at the same time. In this research, ANSYS Workbench is used to analyze force and deformation of the sponge and the fiber. The parameters of the optical fiber were set according to quartz glass. The Young’s modulus of optical fibers was set to 72 GPa, and the Poisson’s ratio was set to 0.15. The parameters of the sponge were measured using a tensile strength machine, as shown in Figure 4. The Young’s modulus was tested to be 32.05 kPa and the Poisson’s ratio was measured to be 0.32.

**Figure 5:**
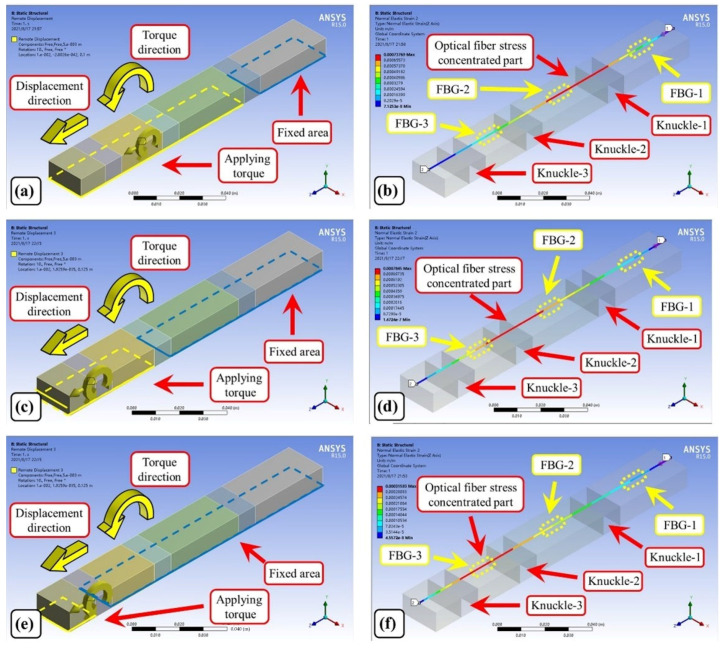
Force analysis of optical fiber during knuckle bending. (**a**) Knuckle-1 bending simulation; (**b**) Fiber force analysis during knuckle-1 bending; (**c**) Knuckle-2 bending simulation; (**d**) Fiber force analysis during knuckle-2 bending; (**e**) Knuckle-3 bending simulation; and (**f**) Fiber force analysis during knuckle-3 bending.

**Figure 6:**
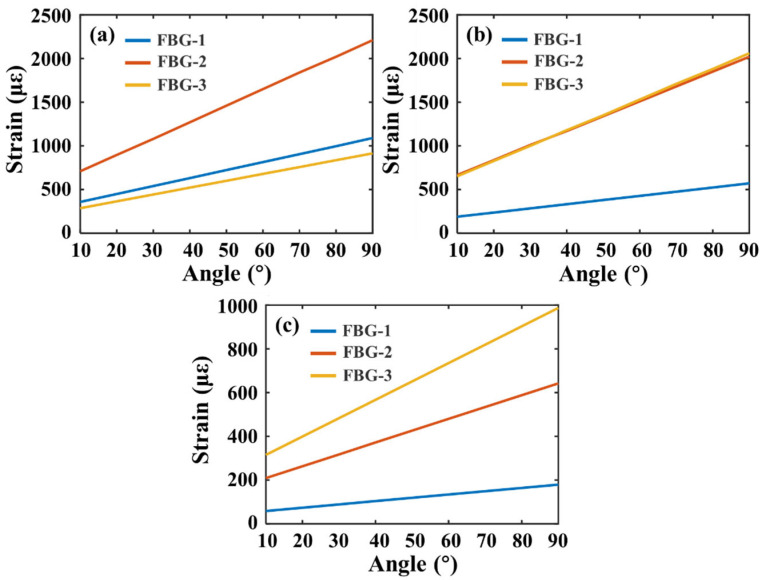
Strain varies with bending angle. (**a**) Strain analysis during knuckle-1 bending; (**b**) Strain analysis during knuckle-2 bending; and (**c**) Strain analysis during knuckle-3 bending.

The finite element model of the sponge and the fiber is shown in Figure 5. The model is analyzed using the “Static Structural” module in ANSYS Workbench. Displacement and torque are applied to the left side of knuckle-1 to simulate the bending of knuckle-1, as shown in Figure 5a. The right side of knuckle-1 is set to “Fixed”. The simulation results are shown in Figure 5b. It can be seen that the strain of the fiber is concentrated near knuckle-1. The FBG-1 and FBG-2, which are closer to knuckle-1, are subjected to a large tensile force, while FBG-3 is subjected to a small tensile force due to the cushioning effect of the sponge. To simulate the bending of knuckle-2, the setting of the force on the sponge is shown in Figure 5c. The results of the corresponding simulation are shown in Figure 5d. The strain of the fiber is concentrated near knuckle-2. The bending of knuckle-2 has a large tensile force on FBG-2 and FBG-3, and a small tensile force on FBG-1. Figure 5e shows the force setting on the sponge when simulating the bending of knuckle-3. From the simulation results in Figure 5f, it can be seen that the tension on fiber FBG-3 is larger than that on FBG-1, and FBG-2 is lower. It can be seen from Figure 6 that the the strain applied to the FBGs is linear during the finger bending. The resolution of strain measurement of FBGs is 0.95 με. According to the analysis of the curve in Figure 6, the worst angle resolution occurs when knuckle-3 is bending. The resolution can be calculated as 0.112°. Compared with a similar method using induction coils [23], which has a resolution of 1.67° when measuring small bending angle, the FBG-based method has a much better resolution. The stress of an FBG can be expressed by its strain, as shown in Equation (3):

(3) σ=Eε

where σ represents stress; E represents the Young’s modulus, which is 72 GPa for optical fiber; and ε represents strain.

Therefore, the stress changes linearly with the strain. It can be seen from Figure 6 that the largest strain occurs when knuckle-1 is bending. The largest strain is 2200 με when the bending angle is 90°. According to Equation (1), the stress will be 158.4 MPa.

The strain limit of FBGs is 17,475 με [24], which is much higher than the strain in this manuscript. Furthermore, the optical fiber used in this research is single-mode optical fibers for communication. In addition to the core and the cladding, the optical fiber is wrapped with a coating layer. The coating layer is composed of acrylate, silicone rubber, and nylon, which ensure it has good elasticity and can protect the optical fiber from being easily broken. The coating layer helps to improve the toughness of the optical fiber. Therefore, the FBGs used in this manuscript can be long-lasting.

## 3. Results

**Figure 7:**
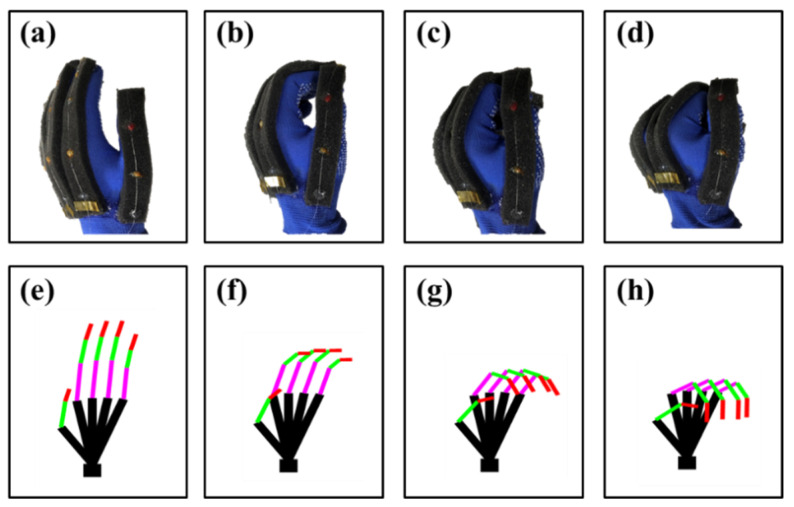
Calibration of finger posture. (**a**) Extension of the palm. (**b**) Slightly extension of the palm. (**c**) Slightly fist of the palm. (**d**) Fist of the palm. (**e**) Model of extension. (**f**) Model of slightly extension. (**g**) Model of slightly fist. (**h**) Model of fist.

The palm was divided into four stages from extension to fist, and the bending of the fingers in each stage was calibrated, as shown in Figure 7. Figure 7a shows the extension of the palm. Figure 7d shows the fist of the palm. Figure 7b,c show the intermediate state of the palm from extension to fist. Figure 7e–h shows the bending fingers models built by matlab. By corresponding the FBGs reflection wavelengths to the finger bending posture and interpolating it using the cubic spline interpolation, the correspondence between the reflected wavelength of FBGs and the finger bending posture is established. The expressions of the cubic spline interpolation are shown in Equations (4) and (5). In this research, the free boundary condition is chosen as shown in Equation (6).

(4) $$S_{i}(x)=a_{i}+b_{i}(x-x_{i})+c_{i}{\left(x-x_{i}\right)}^{2}+d_{i}{\left(x-x_{i}\right)}^{3},\;i=0,1,2,\cdots$$

(5) $$\{\begin{array}{l} a_{i}=y_{i}\\ b_{i}=\frac{y_{i+1}-y_{i}}{h_{i}}-\frac{h_{i}}{2}m_{i}-\frac{h_{i}}{6}(m_{i+1}-m_{i})\\ c_{i}=\frac{m_{i}}{2}\\ d_{i}=\frac{m_{i+1}-m_{i}}{6h_{i}} \end{array},\;i=0,1,2,\cdots$$

(6) $$\left[\begin{array}{ccccccc} 1 & 0 & 0 & 0 & 0 & \cdots & 0\\ h_{0} & 2(h_{0}+h_{1}) & h_{1} & 0 & 0 & \cdots & \vdots \\ 0 & h_{1} & 2(h_{1}+h_{2}) & h_{2} & 0 & \cdots & 0\\ 0 & 0 & h_{2} & 2(h_{2}+h_{3}) & h_{3} & \cdots & 0\\ \vdots & \vdots & \vdots & \vdots & \vdots & \cdots & 0\\ 0 & \cdots & 0 & 0 & 0 & 0 & 1 \end{array}\right]\left[\begin{array}{c} m_{0}\\ m_{1}\\ m_{2}\\ m_{3}\\ \vdots \\ m_{n} \end{array}\right]=6\left[\begin{array}{c} 0\\ \frac{y_{2}-y_{1}}{h_{1}}-\frac{y_{1}-y_{0}}{h_{0}}\\ \frac{y_{3}-y_{2}}{h_{2}}-\frac{y_{2}-y_{1}}{h_{1}}\\ \vdots \\ \frac{y_{n}-y_{n-1}}{h_{n-1}}-\frac{y_{n-1}-y_{n-2}}{h_{n-2}}\\ 0 \end{array}\right]$$

where x represents the angle of finger bending and it is the independent variable; $x_{i}$ represents the measured angle of the *i*-th state; $y_{i}$ represents the measured FBGs reflection value corresponding to $x_{i}$; $S_{i}(x)$ represents the interpolation function for the interval $\left[x_{i},x_{i+1}\right]$; and $h_{i}=x_{i+1}-x_{1}$ represents the step of interpolation.

**Figure 8:**
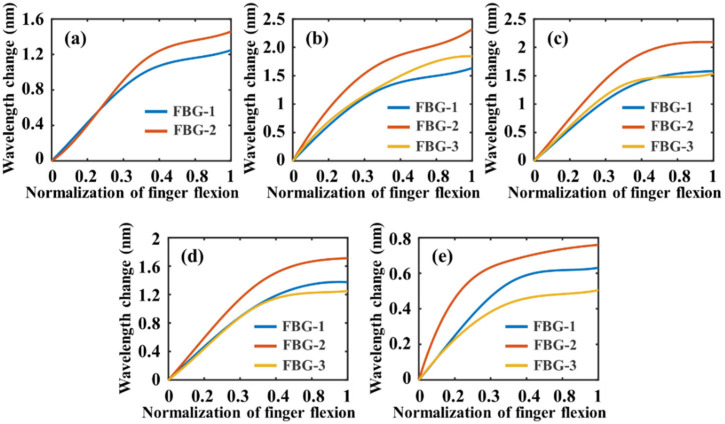
Results of interpolating the reflection peaks of the FBGs using cubic spline interpolation. (**a**) Interpolating of the thumb; (**b**) Interpolating of the index finger; (**c**) Interpolating of the middle finger; (**d**) Interpolating of the fourth finger; and (**e**) Interpolating of the little finger.

**Figure 9:**
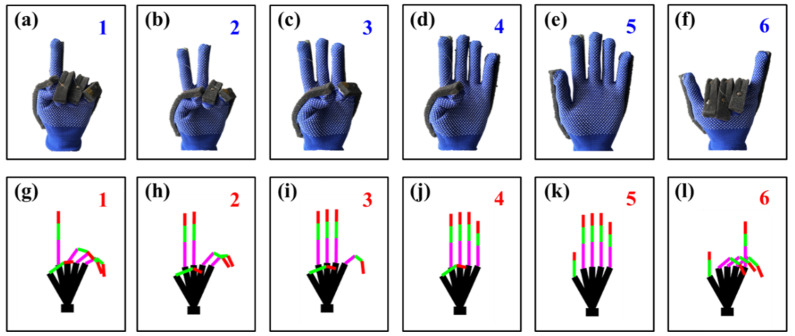
Verification of established finger bending model. (**a**) Hand gesture of 1. (**b**) Hand gesture of 2. (**c**) Hand gesture of 3. (**d**) Hand gesture of 4. (**e**) Hand gesture of 5. (**f**) Hand gesture of 6. (**g**) Model of hand gesture of 1. (**h**) Model of hand gesture of 2. (**i**) Model of hand gesture of 3. (**j**) Model of hand gesture of 4. (**k**) Model of hand gesture of 5. (**l**) Model of hand gesture of 6.

The results of interpolating the reflection peaks of the FBGs using cubic spline interpolation are shown in Figure 8. The horizontal coordinate is the normalization of finger flexion; “0” indicates fully extended fingers and “1” indicates a clenched fist. It can be seen that the FBGs on the same finger have the similar shifts of reflected wavelengths. The wavelength shift of FBGs is approximately linear at the beginning of the finger bending. However, when the fingers approach the fist state, the wavelength shifts of FBGs become non-linear. There are two main reasons for the nonlinearity. First, when subjected to a wide range of stretching and bending at the same time, the FBG exhibits nonlinear effects under complex mechanical and optical effects. In response to this phenomenon, Benjamin Frey et al. [25] presented a solid mechanics model for the thermal and elastic states of a stratified material, considering an embedded optical material domain that represents the FBG. They have provided details of the developed modle on ANSTS. Their developed model is applicable to media of arbitrary shape and composition, including soft matter and materials with nonlinear elasticity and geometric nonlinearity. Second, the occurance of relative sliding between the glove and fingers when the tension of the fiber is large can also cause the nonlinearity. In order to facilitate the application of smart gloves, comprehensively considering those nonlinear factors, the nonlinear curves of FBGs in the measurement process (Figure 7g,h and Figure 9) are determined by calibration. During the calibration, the measurement variance of the FBGs of each finger is 0.24 nm (thumb), 0.31 nm (index finger), 0.20 nm (middle finger), 0.15 nm (the fourth finger), and 0.07 nm (little finger).

To verify the reliability of the established finger bending model, the model is tested as shown in Figure 9. Figure 9a–f represent the hand gestures from 1 to 6, respectively. The smart glove structure shown in Figure 2 was used. A sponge layer was added between the FBGs and the glove as protection. An MOI si155 optical demodulator and the laptop shown in Figure 2 were used to demodulate the wavelength of FBGs. After recording the wavelength change of FBGs with different hand gestures, the curves established in Figure 8 were used to calibrate the bending angle of each knuckle. Then, the the Matlab visualization model was used to show the hand gestures, as shown in Figure 9g–l. From the results, it can be seen that the smart glove based on FBG sensors can correctly recognize the finger postures. The finger movements were repeated in the verification experiment. For example, the action of the thumb in Figure 9a–d repeated one action, and in Figure 9e,f it repeated another action. Therefore, the verification process shown in Figure 9 demonstrates that the the wavelength shift changes of FBGs in this method is reproducible for each finger bending measurement.

## 4. Conclusions

In this research, a smart glove based on FBGs to recognize finger bending posture was proposed. To keep the fiber from being pulled off during finger bending, a 1 cm thick sponge was added between the glove and the fiber to protect the fiber. In the finger posture calibration process, the cubic spline interpolation was used to interpolate the wavelength shift curves of FBGs. The results show that the wavelength shifts of FBGs are approximately linear at the beginning, but the shift becomes non-linear when the finger approaches the fist-clenching state. This is because the relative sliding between the glove and the fingers occurs when the tension of the fiber is large. Finally, the correct response of the cubic spline interpolation for fitting the finger posture was verified by measuring different hand gestures from 1 to 6, and the posture recognition using the smart glove was realized.

## Data Availability

Data sharing is not applicable to this article.
